# The Value of Registration

**Published:** 1917-11-17

**Authors:** 


### NURSING AFFAIRS IN IRELAND.

### The Value of Registration

A CORRESPONDENT of THE HOSPITAL has been good enough to send me an anti-Registration pamphlet, for which I am obliged. But, inasmuch as it is quite out of date, and has already been dissected, I do not propose to discuss it. It must be apparent to the sender and to the author that a vast quantity of water has passed under Westminster Bridge since it was published. Nevertheless there is abundant evidence, including Dr. Kirkpatrick's tract, that every nurse in the kingdom, as well as those interested in nursing affairs, should see precisely what State Registration means and for what it stands.

### A RESULT, AND NOT A CAUSE, OF LEGISLATION.

Now, first of all, Parliament never has passed and never will pass a Registration Act for the protection of a class. The Medical Acts were not enacted for the benefit of the medical profession, and anybody who takes the trouble to peruse an Act of Parliament authorising State Registration will find it clearly stated that the object of the statute is invariably the protection of the public. A case in point is the Midwives Act for Ireland about to be presented to Parliament, framed in the interest of child-welfare and as a factor in stemming the ravages of infant mortality. It happens, as a rule, incidentally, that the State Registered workers of a profession come in for a share of benefit, but this is a result and not a cause of legislation. I do not think it is necessary here to discuss to what extent the public suffer from lack of State Registration of Nurses. The House of Lords has already signified approval of the principle of such an Act being passed. Hence it may be taken for granted that the necessity for protecting the public as well as the nurses is established.

### THE ONLY UNORGANISED PROFESSION.

The question now arises, Will nurses benefit by such legislation, and, if so, how? While strongly combating any such suggestion as that made by Dr. Kirkpatrick that State Registration can or will disqualify untrained persons from nursing for gain, it is abundantly clear that substantial benefit for the profession must almost necessarily ensue. It is an extraordinary anomaly that there is no trade or profession within the British Isles worth mentioning, save and except that of nursing, that remains unorganised. The medical profession is highly organised not alone by its State Register, but by various Royal Colleges of Physicians and Surgeons and by such voluntary Associations as the B.M.A. Nowhere is the need for an organisation of nurses more apparent than in Ireland. The Irish Nurses' Association have on their Committee one trained private nurse to represent private nurses, but this lady is not only not elected by the people whom she is supposed to represent, but neither she nor the Association have any knowledge of the number of private trained nurses working in Ireland, nor have they any means of making an approximately accurate guess. Nobody knows, and nobody has any means of finding out. They are scattered independent units without any common bond or common centre. No such anomaly prevails in any other profession. A doctor, a solicitor, a dentist, a veterinary surgeon practising in the most remote locality can be communicated with at once by consulting the Register. It may appear an easy matter to trace trained nurses working in hospitals, but it is not. There is no register anywhere which shows even the number of such nurses, not to mention their names. Doubtless the Local Government Board could form a list showing the names and qualifications of Poor-Law nurses, but it would not be a public document, and the same applies to Jubilee nurses.

The result of this condition of affairs is that the nursing profession is inarticulate. Nor can it receive a message. It ought not to be difficult to realise from what enormous disadvantages a deaf mute suffers. Every possible artifice that science and skill can devise is provided in order to redress the wrongs of Nature, and yet there is a great gulf fixed between him and the man who is blessed with the possession of all his faculties.

### THE WALLS OF JERICHO.

From the nurses' point of view, that with which these remarks are alone concerned, the supreme value of State Registration would consist in the organisation of the profession, by which they could, if they found it necessary at any time, march round the walls of Jericho, and with a great shout cause them to tumble down. Let the nurses of the United Kingdom take a leaf from the doctors' book. Has it been forgotten with what *esprit de corps* they fought when the National Health Insurance Act was being passed? This is an isolated example of a force that never rests. Every wind that blows, so to speak, is watched with jealous care to see that the interests of the profession are safeguarded and, if possible, benefited. A great deal of legislation concerning public health has taken place, and much more will follow. The interests of the trained nurse are bound up with all such movements, but she stands by helpless and dumb.

It may be pointed out that for the purpose described any complete Register would suffice, and this is true. But can such a Register by any device be obtained? I do not think so. There are, I am informed, some 70,000 trained nurses in the kingdom, and of these only a tithe have joined the College of Nursing, whose roll is by a long way the largest in existence. If State Registration were law, the whole 70,000 names would appear on the Register, and it would be obviously within their power to achieve a great deal which would make for material benefit.

### A BUSINESS AFFAIR OF THE FIRST MAGNITUDE.

In conclusion, may I point out that legislation may be long delayed? In the best of circumstances—that is to say, if the entire profession were united—it must be remembered that we are in the throes of war, and the nation's effort must be concentrated on the task of winning it, so that all Parliamentary activity is necessarily subordinate. But if opposition is offered from any quarter, delay may be indefinite. Hence the utter folly of the nurses of the three kingdoms in failing without delay to do that which actually lies at their door, which they can accomplish for themselves without any Parliamentary assistance. I refer to joining the membership of the College of Nursing. I have no personal reason whatever to make such a recommendation, but I see in it a potential means of organising the whole profession not only of these countries, but also of the entire Empire, and if State Registration never came, such a force of public nursing opinion would be resistless. This is not a matter of sentiment, but a business affair of the first magnitude.

